# Oleamide activates peroxisome proliferator-activated receptor gamma (PPARγ) *in vitro*

**DOI:** 10.1186/1476-511X-11-51

**Published:** 2012-05-14

**Authors:** Stephen PH Alexander, Andrew J Bennett

**Affiliations:** 1 FRAME Laboratory, University of Nottingham Medical School, Nottingham, NG7 2UH, England; 2 School of Biomedical Sciences, University of Nottingham Medical School, Nottingham, NG7 2UH, England; 3 Current address: Istituto Italiano di Tecnologia, via Morego, Genova, Italy

**Keywords:** Oleamide, Peroxisome proliferator, PPAR, Endocannabinoids

## Abstract

**Background:**

Oleamide (ODA) is a fatty acid primary amide first identified in the cerebrospinal fluid of sleep-deprived cats, which exerts effects on vascular and neuronal tissues, with a variety of molecular targets including cannabinoid receptors and gap junctions. It has recently been reported to exert a hypolipidemic effect in hamsters. Here, we have investigated the nuclear receptor family of peroxisome proliferator-activated receptors (PPARs) as potential targets for ODA action.

**Results:**

Activation of PPARα, PPARβ and PPARγ was assessed using recombinant expression in Chinese hamster ovary cells with a luciferase reporter gene assay. Direct binding of ODA to the ligand binding domain of each of the three PPARs was monitored in a cell-free fluorescent ligand competition assay. A well-established assay of PPARγ activity, the differentiation of 3T3-L1 murine fibroblasts into adipocytes, was assessed using an Oil Red O uptake-based assay. ODA, at 10 and 50 μM, was able to transactivate PPARα, PPARβ and PPARγ receptors. ODA bound to the ligand binding domain of all three PPARs, although complete displacement of fluorescent ligand was only evident for PPARγ, at which an IC_50_ value of 38 μM was estimated. In 3T3-L1 cells, ODA, at 10 and 20 μM, induced adipogenesis.

**Conclusions:**

We have, therefore, identified a novel site of action of ODA through PPAR nuclear receptors and shown how ODA should be considered as a weak PPARγ ligand *in vitro*.

## Background

Oleamide (ODA, (Z)-octadec-9-enamide) is a fatty acid primary amide first identified as an endogenous lipoamide in the cerebrospinal fluid of sleep-deprived cats [1]. The ODA biosynthesis pathway has not been intensively investigated, but it has been hypothesised to involve the peptidyl glycine α-amidating monooxygenase [2]. Alternatively, Driscoll et al. [3] suggested that cytochrome c may also be a route for ODA synthesis. ODA catabolism appears to be primarily due to hydrolysis by fatty acid amide hydrolase (FAAH) to oleic acid and ammonia [2,4]. More recently, a second hydrolase, restricted to a limited number of higher mammals, named FAAH-2 with overlapping, but distinct, tissue distribution has been discovered, at which ODA appears to be the preferred substrate [5].

*In vivo* administration of ODA has a variety of observable effects: as a sleep-inducing factor [6], also eliciting hypothermia, analgesia and hypo-locomotion [7]. Extended dietary administration of ODA *in vivo* caused hypolipidemia in high fat-fed hamsters through an undetermined mechanism [8]. *In vitro*, ODA has also been reported to induce vasorelaxation in the rat small mesenteric artery [9]. While the biological effects of ODA are well documented, the molecular mechanisms and site of action remain elusive. *In vitro*, ODA can inhibit gap junction formation [10], modulate GABA [11] and 5-HT [12] receptors and bind to CB_1_ cannabinoid receptors [13].

Peroxisome proliferator-activated receptors (PPARs) are a subfamily of nuclear receptors, which function as ligand-activated transcription factors [14,15]. There are three different PPAR isotypes, PPARα, PPARβ or PPARγ [16] encoded by distinct genes and linked to lipid metabolism and glucose homeostasis, as well as inflammation and cytoprotection. Endogenous ligands for PPARs include fatty acids and their metabolites, including prostaglandins and leukotrienes [14,15]. A variety of cannabinoids and related molecules have been reported to bind and activate PPARs and at least some of their actions might be explained by this interaction [17,18]. We hypothesized that the hypolipidemic effects of ODA observed *in vivo*[8] might be explained by activity at PPARs and so we have tested the ability of ODA to occupy and transactivate PPARs *in vitro.*

## Results

### PPAR transactivation

**Figure 1:**
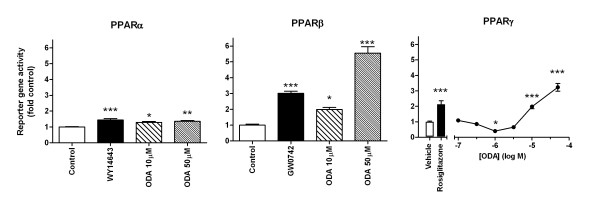
**Assay of reporter gene activity in PPARα-, PPARβ- and PPARγ-transiently transfected Chinese hamster ovary cells.** Positive controls at PPARα, PPARβ and PPARγ were WY14643 (10 μM), GW0742 (1 μM) and rosiglitazone (1 μM), respectively, with DMSO (0.1%) as a vehicle control. Data are means ± SEM from six different experiments conducted in duplicate. * *P* < 0.05, ** *P* < 0.01, *** *P* < 0.001 assessed using one-way ANOVA with Bonferroni’s post hoc test.

ODA at 10 and 50 μM evoked significant activation of PPARα, PPARβ and PPARγ receptors in CHO cells over-expressing these nuclear receptors (Figure 1). ODA appeared to have the most marked, concentration-dependent effects on PPARβ and PPARγ receptors, with 50 μM ODA evoking responses in PPARβ- and PPARγ-expressing cells to 5.5-fold and 3.2-fold control, respectively (Figure 1).

### PPAR occupancy assays

**Figure 2:**
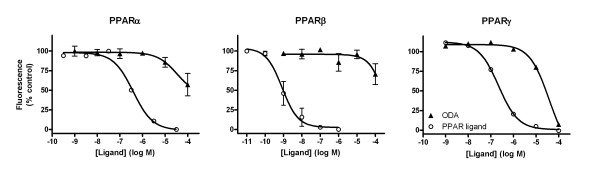
**Assay of fluorescent ligand binding to ligand binding domains of PPARα, PPARβ and PPARγ.** PPAR ligands employed as positive controls alongside ODA were WY14643, GW0742 or rosiglitazone for PPARα, PPARβ and PPARγ, respectively. Data are means ± range of two experiments (except WY14643, n = 1) conducted in sextuplicate.

Since whole cell reporter gene assays may be complicated by metabolic conversion of putative ligands, we then tested whether ODA was able to bind directly to PPARs, using selective ligands for each receptor as positive controls. In each case, the positive control evoked concentration-dependent inhibition of fluorescent ligand binding to the ligand binding domains of PPARα, PPARβ and PPARγ (Figure 2). Thus, WY14643, GW0742 and rosiglitazone exhibited potencies (IC_50_ values) of 3.8 × 10^-7^, 8.4 × 10^-10^ and 2.2 × 10^-7^ M at PPARα, PPARβ and PPARγ, respectively.

In comparison, although ODA evoked concentration-dependent displacement of fluorescent ligand from each receptor binding site, displacement at PPARα and PPARβ was incomplete at the highest concentration employed (100 μM). The potency of ODA at PPARγ was estimated at 3.8 x 10^-5^ M (Figure 2).

### 3T3-L1 differentiation

**Figure 3:**
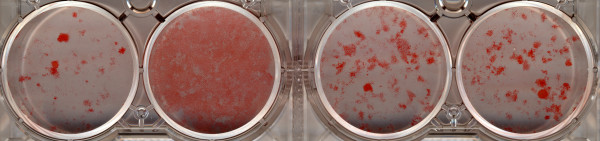
**Oil Red O staining following differentiation of 3T3-L1 cells into adipocytes in the presence of (from left to right) vehicle (0.1% DMSO), rosiglitazone (10 μM), ODA (10 μM) or ODA (20 μM).** The picture is representative of three replicates.

In the Oil Red O uptake-based assay, the positive control rosiglitazone (10 μM) was confirmed to increase markedly the number of 3T3-L1 cells stained by the lipid-sensitive dye (Figure 3). ODA (10–20 μM) was also able to induce differentiation of 3T3-L1 cells into adipocytes compared to the vehicle-treated controls, although the extent of differentiation was less marked than in the presence of rosiglitazone (Figure 3).

## Discussion

In this study, we show that a further endocannabinoid-like molecule, oleamide, is able to occupy and activate PPAR nuclear receptors. As well as the phytocannabinoids THC [19], the major psychoactive ingredient in *Cannabis*, and cannabidiol [20], a number of endogenous cannabinoids have been shown to activate PPARs. In particular, anandamide, virodhamine, *N*-arachidonoyldopamine, noladin and 2-arachidonoylglycerol, as well as *N*-oleoylethanolamine and *N*-palmitoylethanolamine, have been shown to activate various members of the PPAR family [17,18].

Our results show that concentrations of ODA in the mid-micromolar range were able to transactivate PPARα, PPARβ and PPARγ nuclear receptors and this effect was demonstrated to be concentration-dependent for PPARβ and γ (Figure 1B and C). Interestingly, lower concentrations of ODA (at 1 μM) appeared to significantly inhibit PPARγ basal activation. This effect might be explained by high basal activation of these receptors by endogenous ligands in CHO cells, for example as components of the serum. A similar effect has been observed for the dietary polyunsaturated fatty acids, docosahexaenoic acid and eicosapentaenoic acid, which act as agonists at PPARs, but reduce the tonic activation apparently evoked by endogenous higher affinity agonists [21].

We were able to show directly that ODA was able to occupy the ligand binding domain of all three receptors, implying that the enzymatic generation of oleic acid from ODA is not a simple explanation for the observed effects. Of the three subtypes of PPAR, the potency of ODA appeared highest at PPARγ (Figure 2), although functional effects on PPARβ appeared higher in reporter gene assays (Figure 1). This may be attributed to greater amplification of PPARβ-evoked responses, or alternatively, to background levels of PPARγ activity that were relatively elevated.

The IC_50_ value (38 μM) of ODA at the PPARγ ligand binding site is slightly higher than other endocannabinoid-like molecules reported to activate PPARγ. IC_50_ values for anandamide and 2-arachidonoylglycerol at PPARγ are reported to be around 10 μM [22]. The phytocannabinoid cannabidiol was reported to have a potency of around 5 μM at PPARγ, while the IC_50_ value for ajulemic acid was around 600 nM [20]. However, other saturated or unsaturated fatty acids that are regarded as endogenous ligands for the three different PPARs isotypes all have micromolar affinities to these receptors, in line with their serum levels [14,15].

In order to address the issue of whether ODA might be a physiological agonist at PPARγ, we investigated the phenomenon of adipogenesis, which is a well recognised consequence of PPARγ activation [14,15]. Using 3T3-L1 cells, we observed that ODA at 10–20 μM was able to induce differentiation into adipocytes (Figure 3).

The proposed novel site of action for ODA through PPAR activation might be involved in some of the previously reported ODA effects. ODA has been reported to induce vasorelaxation in the rat small mesenteric artery *in vitro* through activation of an undefined receptor which may be coupled to Ca^2+^-sensitive K^+^ channels and G_i/o_ proteins [9]. However, the mechanism by which it elicits vasorelaxation has not been fully explained. Central effects of *N*-oleoylethanolamine (OEA) and *N-*palmitoylethanolamine, two other endocannabinoid-related molecules, have been demonstrated to be mediated through PPARα activation [23-26]. OEA has also been reported to elicit loss of appetite and to reduce body weight gain in mice with a mechanism dependent on PPARα [23]. Moreover, we previously showed that OEA pre-treatment reduced infarct volume from middle cerebral artery occlusion in wild-type, but not in PPARα-null, mice [27]. In these two latter studies, OEA was shown to bind to the ligand binding domain of PPARα and to transactivate both PPARα and PPARβ, without activating PPARγ. Our data show that ODA binds to all three PPAR LBDs, with apparently highest affinity for PPARγ (Figure 2). OEA and ODA share the same fatty acid chain, oleic acid, which, together with a variety of other saturated and unsaturated fatty acids, is one of the natural ligands of PPARα. By contrast, PPARγ exhibits greater selectivity than PPARα and is usually activated by polyunsaturated fatty acids [14,15]. Even if the limiting factor for PPAR binding is usually the length and saturation level of the fatty acid chain, in this case the head residue would appear to confer selectivity for PPARγ binding between OEA and ODA. However, no direct evidence is available in the literature of OEA binding (or not) to the ligand binding domain of PPARγ.

The most relevant *in vivo* observation that might correlate with ODA’s agonist activity at PPARs is the hypolipidemic effect when administered in the diet to high fat-fed hamsters [8]. In this report, ODA dose-dependently reduced plasma triglyceride levels, as well as LDL cholesterol and liver triglyceride, without altering HDL cholesterol levels. This appears to be consistent with a site of action through PPARs, at least in part, since agonists at both PPARα and γ are observed to increase HDL cholesterol levels, whilst reducing LDL cholesterol, as well as plasma and liver triglycerides [15].

## Conclusions

In summary, we have identified a novel site of action of ODA, through PPARs. Our data indicate that ODA can be regarded as a low affinity pan-PPAR ligand *in vitro,* being able to transactivate all three isotypes of this nuclear receptor family. ODA appeared to be most potent as a ligand of PPARγ.

## Methods

### Chemicals

ODA was prepared by condensation of oleoyl chloride (Sigma Chemical Company, Poole, UK) with saturated ammonia solution (Fisher Scientific, Loughborough, UK). Following purification over silica, TLC and NMR analysis indicated a single product with undetectable levels of oleic acid. WY14643 and GW0742 were purchased from Tocris Cookson (Bristol, UK), while rosiglitazone was a kind donation from GlaxoSmithKline (UK).

### Cell culture

CHO cells (originally obtained from ECACC, Salisbury, UK) were incubated at 37°C and 5% CO_2_ in Dulbecco’s Modified Medium (DMEM, Sigma, Poole, UK) supplemented with 10% (v/v) heat-inactivated fetal bovine serum (FBS, Sigma, Poole, UK), 2 mM L-glutamine (Sigma, Poole, UK) and streptomycin/penicillin (50 μg ml^-1^ and 50 U ml^-1^, respectively, Sigma, Poole, UK).

### Transactivation assay

A luciferase reporter construct under the control of 3xPPRE was transfected into CHO cells, together with a pcDNA3.1 plasmid expressing the human PPARα, PPARβ or PPARγ2 gene. Transient transfection of CHO cells was carried out by the polyethyleneimine method with the ratio nitrogen (N) to DNA phosphate (P) of N/P = 15 as previously described [27]. 4 hours after transfection, CHO cells were treated with either vehicle control (0.1% DMSO) or ligand, as indicated. 24 hours after treatment, cells were harvested and lysed with Passive Lysis Buffer (Promega, Wisconsin, USA) and luciferase expression was monitored using the Luciferase Assay System (Promega, Wisconsin, USA) and a luminometer (TD-20/20, Turner Biosystems, California, USA). Data were calculated as Relative Luciferase Units (RLU) mg^-1^ protein and expressed as fold activation compared to control.

### Protein quantification

Protein concentrations of the samples from the reporter gene assay were titrated using Bio-Rad protein assay (Bio-Rad, California, USA).

### PPAR occupancy assays

Binding experiments to PPARα and PPARβ were carried out with Lanthascreen^™^ TR-FRET PPAR Competitive Binding Assays (Invitrogen, California, USA) following the manufacturer’s instructions. For PPARγ occupancy, binding experiments were carried out with Polarscreen^™^ PPAR Competitor Assay Green (Invitrogen, California, USA) following the manufacturer’s instructions. The kits use a fluorescent small molecule pan-PPAR ligand (20nM for PPARα-β and 2.5nM for PPARγ) and human-PPAR ligand binding domains. Fluorescent signal (340 nm excitation and 495–520 nm emission; PPARα-β) and fluorescence polarization (485 nm excitation and 535 nm emission; PPARγ) were measured at room temperature in black 384-well plates using an Envision MultiLabel plate reader. Data are reported as percentage of the control.

### 3T3-L1 differentiation

3T3-L1 cells (originally obtained from ECACC, Salisbury, UK) were grown to confluence in 6-well plates. 48 hours after confluence, the culture medium was replaced and supplemented with 1 μM dexamethasone and 5 μg mL^-1^ insulin (Sigma). After 48 hours, the culture medium was replaced and supplemented with 5 μg mL^-1^ insulin and vehicle (0.1% DMSO) or ligand, as indicated. Cells were grown for around 10 days, checking for differentiation and changing the medium 2–3 times per week. Once differentiation occurred, cells were treated for 10 minutes with 4% formalin and incubated with Oil Red O at room temperature for 1 hour. Images were taken from the bottom of the wells with a scanner (Epson).

### Statistical analysis

Statistical differences among treatments were assessed using one-way ANOVA with Bonferroni’s post hoc test. Displacement curves were fitted to the data using a one-site competition binding model (GraphPad Prism, California, USA).

## Abbreviations

FAAH: Fatty acid amide hydrolase; LBD: Ligand binding domain; ODA: Oleamide; OEA: N-oleoylethanolamine; PPAR: Peroxisome proliferator-activated receptors; THC: Δ9-tetrahydrocannabinol.

## Competing interests

The authors declare that they have no competing interests.

## Authors’ contributions

All authors participated in the design and co-ordination of the study. MD conducted the reporter gene, receptor binding and differentiation assays, and drafted the manuscript. All authors participated in the analysis of the data and read and approved the final manuscript.
